# Crystal structure studies of 4-ethyl­piperazin-1-ium 3,5-di­nitro­benzoate, 4-methyl­piperazin-1-ium 3,5-di­nitro­benzoate and 4-methyl­piperazin-1-ium 4-iodo­benzoate

**DOI:** 10.1107/S2056989021010689

**Published:** 2021-10-21

**Authors:** Sriramapura D. Archana, Haruvegowda Kiran Kumar, Hemmige S. Yathirajan, Sabine Foro, Mohammed S. M. Abdelbaky, Santiago Garcia-Granda

**Affiliations:** a Department of Studies in Chemistry, University of Mysore, Manasagangotri, Mysore-570 006, India; b Institute of Materials Science, Darmstadt University of Technology, Alarich-Weiss-Strasse 2, D-64287 Darmstadt, Germany; c Department of Physical and Analytical Chemistry, Faculty of Chemistry, Oviedo University-CINN, Oviedo 33006, Spain

**Keywords:** crystal structure, piperazinium salts, benzoate anion, biological activity

## Abstract

As part of our ongoing investigation on the chemical and biological properties of piperazinium salts, we synthesized three novel compounds: 1-ethyl­piperazinium 3,5-di­nitro­benzoate (I), 1-methyl­piperazinium 3,5-di­nitro­benzoate (II) and 1-methyl­piperazinium 4-iodo­benzoate (III). The crystal structures of these compounds are built up of organic layers formed by the strong connection between the mol­ecules by hydrogen bonds of type N—H⋯O. These layers are linked through N—H⋯O hydrogen bonds and C—H⋯O inter­actions or C—I⋯N halogen bonding, leading to the formation of a three-dimensional network.

## Abstract 2

Three novel piperazinium salts are described. Their crystal structure is based on layers formed by hydrogen bonding, halogen bonding and other weak inter­actions. One exhibits an asymmetric unit containing a 1-ethyl­piperazinium cation and a 3,5-di­nitro­benzoate anion while the other two salts have asymmetric units containing 1-methyl­piperazinium as a common cation and a 3,5-di­nitro­benzoate anion or a 4-iodo­benzoate anion.

## Chemical context

Piperazines and substituted piperazines are important pharmacophores that can be found in many biologically active compounds across a number of different therapeutic areas (Berkheij, 2005 ▸) such as anti­fungal (Upadhayaya *et al.*, 2004 ▸), anti-bacterial, anti-malarial and anti-psychotic agents (Choudhary *et al.*, 2006 ▸). A valuable insight into recent advances on anti­microbial activity of piperazine derivatives has been reported (Kharb *et al.*, 2012 ▸).

Piperazines are among the most important building blocks in today’s drug discovery efforts and are found in biologically active compounds across a number of different therapeutic areas (Brockunier *et al.*, 2004 ▸; Bogatcheva *et al.*, 2006 ▸). A review of the current pharmacological and toxicological information for piperazine derivatives is given by Elliott (2011 ▸).

1-Ethyl­piperazine is used in the synthesis of 2-{2-meth­oxy-5-[(4-methyl­piperazin-1-yl)sulfon­yl]phen­yl}-1*H*-benzo[*d*]imid­azole hydro­chloride and 2-{5-[(4-ethyl­piperazin-1-yl)sulfon­yl]-2-meth­oxy­phen­yl}-1*H*-benzo[*d*]imidazole hydro­chloride as benzimidazole analogs of sildenafil, which is marketed for the treatment of erectile dysfunction (Qandil, 2012 ▸). It is also employed as an inter­mediate in veterinary medicine and serves as a precursor in the preparation of dyes. *N*-Ethyl piperazine is used in the synthesis of enrofloxacin, which is an anti­biotic used to treat bacterial infections. It is also used in the synthesis of dyes, agrochemicals and other pharmaceutical compounds. The crystal structures of compounds derived from 1-ethyl­piperazine, *viz*., chloro­bis­(2-chloro­benz­yl)(4-eth­ylpiperazine-1-di­thio­carbamato-κ^2^ *S*,*S*′)tin(IV) (Li & Li, 2007 ▸), 1-diphen­ylmethyl-4-eth­ylpiperazine-1,4-diium dichloride (Qiao *et al.*, 2010 ▸), (*S*)-3-chloro-4-(4-eth­ylpiperazin-1-yl)-5-[(1*R*,2*S*,5*R*)-2-isopropyl-5-meth­ylcyclo­hex­yloxy]furan-2(5*H*)-one (Fu *et al.*, 2010 ▸), 4-{[5-(4-chloro­phen­yl)-1-(4-fluoro­phen­yl)-1*H*-pyrazol-3-yl]carbon­yl}-*N*-eth­ylpiperazine-1-carboxamide (Shahani *et al.*, 2011 ▸), 2-[4-(2-meth­oxy­phen­yl)piperazin-1-yl]-*N*-(pyridin-2-yl)acet­amide (Lu & Jiang, 2011 ▸), *N*-(4-chloro­phen­yl)-4-eth­ylpiperazine-1-carboxamide (Li, 2011 ▸) and tri­chlorido­(1-eth­ylpiperazin-1-ium)cobalt(II) (Dhieb *et al.*, 2014 ▸) have been reported.

1-Methyl­piperazine is used in the preparation of 2-(4-methyl-1-piperazinylmeth­yl)acrylo­phenone as an anti­microtubular drug (Mallevais *et al.*, 1984 ▸). It is involved in the preparation of 1-(4-meth­oxy­phen­yl)-4-methyl­piperazine by reaction with 1-chloro-4-meth­oxy-benzene. It acts as an inter­mediate in the synthesis of active pharmaceutical ingredients such as ofloxacin, rifampicin, clozapine, sildenafil, trifluoperazine and zopiclone. The crystal structures of 1-meth­ylpiperazine-1,4-diium 4-nitro­phthalate(2−) 4-nitro­phthalic acid monohydrate (Guo, 2004 ▸), (−)-2-methyl­piper­azin-1-ium perchlorate (Peng, 2010 ▸), 1-methyl­piperazine-1,4-diium dipicrate (Dutkiewicz *et al.*, 2011 ▸), 1-meth­ylpiperazine-1,4-dium bis­(hydrogen oxalate) (Essid *et al.*, 2014 ▸), 2-meth­ylpiperazine-1,4-diium bis­(hydrogen maleate) (Wecharine *et al.*, 2015 ▸) and 2-methyl­piperazine-1,4-diium bis­(hydrogen maleate) (Wecharine & Arto, 2015 ▸), have been reported. 
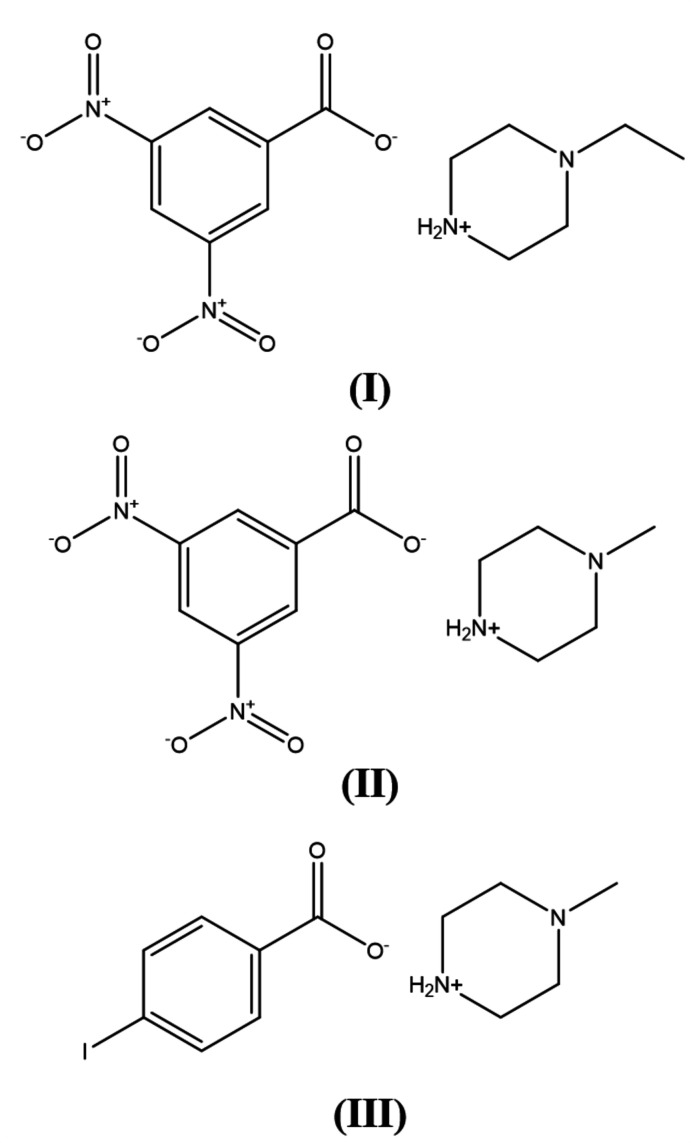


We have recently reported the crystal structures of some salts of 4-meth­oxy­phenyl­piperazine (Kiran Kumar *et al.*, 2019 ▸) and also 2-meth­oxy­phenyl­piperazine (Harish Chinthal *et al.*, 2020 ▸). In view of the importance of piperazines in general and the use of 1-eth­yl/methyl­piperazine in particular, the present paper reports the crystal structure of salts 1-ethyl­piperazinium 3,5-di­nitro­benzoate (I), 1-methyl­piperazinium 3,5-di­nitro­benzoate (II) and 1-methyl­piperazinium 4-iodo­benzoate (III).

## Structural commentary

**Figure 1:**
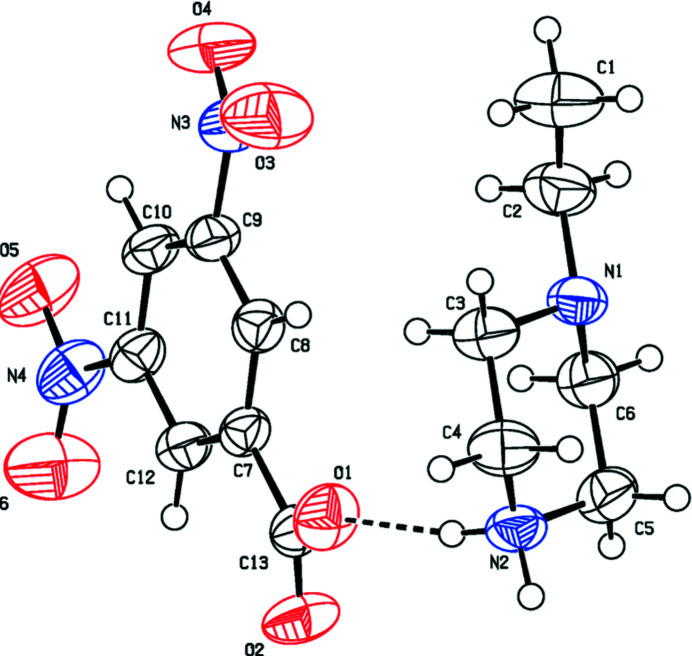
The mol­ecular structure of compound (I), showing the atom labelling. Displacement ellipsoids are drawn at the 50% probability level.

The mol­ecular structures of the title salts (I), (II) and (III) are illustrated in Figs. 1 ▸, 2 ▸ and 3 ▸, respectively. The asymmetric unit of compound (I) is composed of one 1-ethyl­piperazinium cation and one 3,5-di­nitro­benzoate anion while (II) consists of a 1-methyl­piperazinium cation and a 3,5-di­nitro­benzoate anion. Compound (III) crystallizes with one 1-methyl­piperazinium cation and one 4-iodo­benzoate anion in the asymmetric unit. In all compounds, the piperazine rings have a chair conformation with a positively charged protonated N atom with a maximum deviation from their mean plane of 0.239 (2), 0.258 (2) and 0.238 (2) Å at atom N1, for the three title compounds, respectively. The benzene rings are almost planar, with maximum deviations of 0.010 (2), 0.006 (2) and 0.006 (3) Å at atoms C8, C10 and C8 for (I), (II) and (III) respectively. The substituents of the benzene rings in all compounds are approximately in the same plane and do not deviate significantly from planarity.

## Supra­molecular features

**Figure 4:**
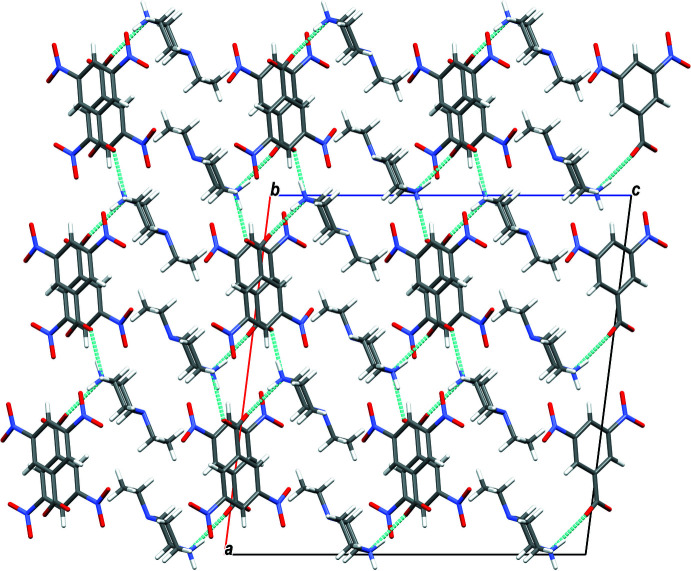
Mol­ecular packing of (I) with hydrogen bonding shown as dashed lines.

**Figure 5:**
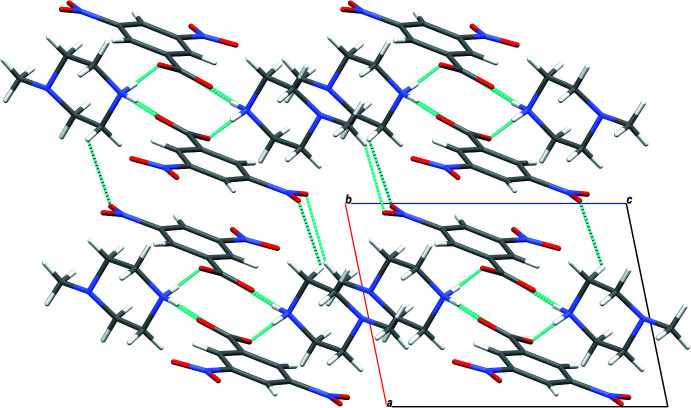
Mol­ecular packing of (II) with hydrogen bonding shown as dashed lines..

**Figure 6:**
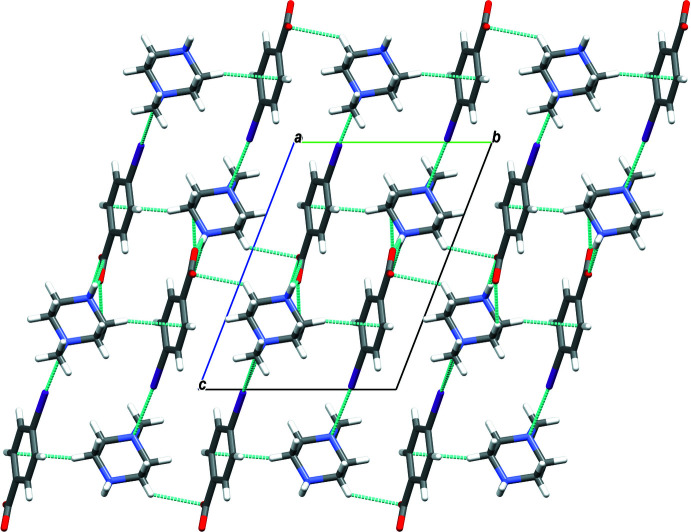
Mol­ecular packing of (III) with hydrogen bonding shown as dashed lines..

In the crystal of (I), the cation and anion are linked by N2—H21⋯O1 hydrogen bonds, forming layers extending along the *c*-axis direction. The layers are connected *via* N2—H22⋯O2 hydrogen bonds, forming sheets lying parallel to the *ac* plane (Table 1 ▸ and Fig. 4 ▸). The crystal structure of compound (II) is built up of N2—H21⋯O2 and N2—H22⋯O1 hydrogen bonds that connect the mol­ecules in strong layers along the *c*-axis direction. The layers are linked *via* weak inter­actions of the type C—H⋯O, giving a three-dimensional network along the *b* axis (Table 2 ▸ and Fig. 5 ▸). The mol­ecules in the crystal of (III) are linked by N2—H21⋯O2, N2—H22⋯O1, C—H⋯O and C—H⋯π inter­actions, forming layers along the *b* axis. The layers are linked through C—I⋯N halogen bonding with C9—I1 and I1⋯N1(1 − *x*, 1 − *y*, −*z*) bond distnces of 2.103 (2) and 3.073 (2) Å, respectively, and bond angle of 174.33 (8)°, leading to a three-dimensional structure (Table 3 ▸ and Fig. 6 ▸).

## Database survey

A search of the Cambridge Structural Database (Version 2020.3.0, last update March 2021; Groom *et al.*, 2016 ▸) for the piperazinium cation and benzoate anion involved in the three salts gave 62 hits, 60 of which have branched aromatic substituents either on the piperazinium cation, the benzoate anion or both, that make their structures extremely different from those of the title salts. The other two compounds are quite similar to the title mol­ecules: 4-meth­ylpiperazin-1-ium 2-amino-5-iodo­benzoate (MAVMEC: Zhu & Guo, 2005 ▸) and 1-meth­ylpiperazine-1,4-diium 4-nitro­phthalate(2-) 4-nitro­phthalic acid monohydrate (IZEFY: Guo, 2004 ▸), which share the cationic part and its chair conformation with salts (II) and (III). The crystal structures of the two compounds are based on differently sized rings formed through hydrogen-bond contacts, which then aggregate into a 3D framework.

## Synthesis and crystallization

For the synthesis of (I), a solution of commercially available 1-ethyl­piperazine (100 mg, 0.88 mol) (from Sigma-Aldrich) in methanol (10 ml) was mixed with an equimolar solution of 3,5-di­nitro­benzoic acid (186.6 mg, 0.88 mol). Compounds (II) and (III) were prepared by the same method in which 1-methyl­piperazine (100 mg, 1.0 mol) in methanol (10 ml) was mixed with an equimolar solution of 3,5-di­nitro­benzoic acid (212 mg, 1.0 mol) for (II) or with an equimolar solution of 4-iodo­benzoic acid (248 mg, 1.0 mol) for (III). The corresponding mixtures were stirred for 30 min at 323 K and allowed to stand at room temperature. X-ray quality crystals were formed upon slow evaporation in a week time (m.p. 453–455 K, 459–461 K and 410–412 K, respectively).

## Refinement

Crystal data, data collection and structure refinement details are summarized in Table 4 ▸. The H atoms bound to C were positioned with idealized geometry and refined using a riding model with aromatic C—H = 0.93 Å, 0.96 Å (meth­yl) or 0.97 Å (methyl­ene). The H atoms of the N atom were located in a difference map and later restrained to the distance N—H = 0.86 (2) Å. All H atoms were refined with isotropic displace­ment parameters set at 1.2*U*_eq_ (C-aromatic, C-methyl­ene, N) or 1.5*U*_eq_ (C-meth­yl) of the parent atom.

## Supplementary Material

Crystal structure: contains datablock(s) global, I, II, III. DOI: 10.1107/S2056989021010689/dj2037sup1.cif

Structure factors: contains datablock(s) I. DOI: 10.1107/S2056989021010689/dj2037Isup2.hkl

Structure factors: contains datablock(s) II. DOI: 10.1107/S2056989021010689/dj2037IIsup3.hkl

Structure factors: contains datablock(s) III. DOI: 10.1107/S2056989021010689/dj2037IIIsup4.hkl

Supporting information file. DOI: 10.1107/S2056989021010689/dj2037Isup5.cml

Supporting information file. DOI: 10.1107/S2056989021010689/dj2037IIsup6.cml

Supporting information file. DOI: 10.1107/S2056989021010689/dj2037IIIsup7.cml

CCDC references: 2115865, 2115864, 2115863

Additional supporting information:  crystallographic information; 3D view; checkCIF report

## Figures and Tables

**Figure 2:**
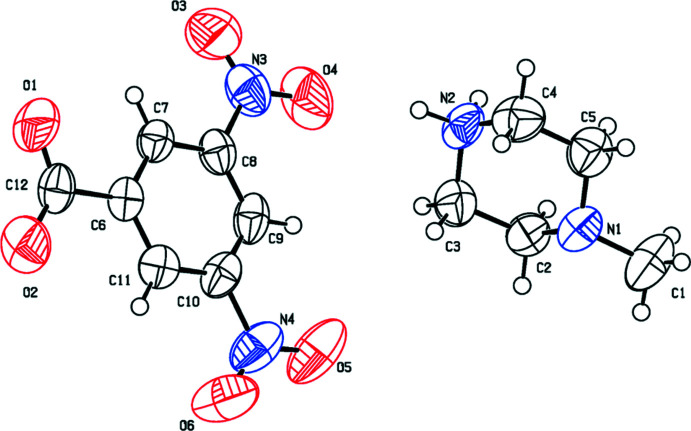
The mol­ecular structure of compound (II), showing the atom labelling. Displacement ellipsoids are drawn at the 50% probability level.

**Figure 3:**
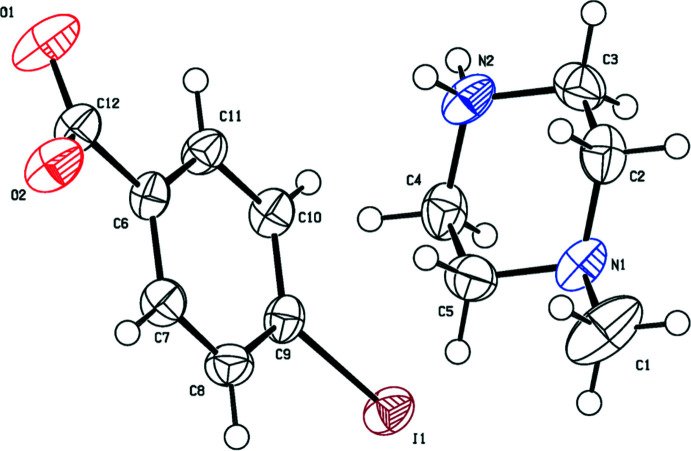
The mol­ecular structure of compound (III), showing the atom labelling. Displacement ellipsoids are drawn at the 50% probability level.

**Table 1:** Hydrogen-bond geometry (Å, °) for (I)

*D*—H⋯*A*	*D*—H	H⋯*A*	*D*⋯*A*	*D*—H⋯*A*
N2—H21⋯O1	0.91 (2)	1.88 (2)	2.768 (2)	168 (3)
N2—H22⋯O2^i^	0.92 (2)	1.77 (2)	2.684 (2)	171 (3)

Symmetry code: (i) -x, -y, -z.

**Table 2:** Hydrogen-bond geometry (Å, °) for (II)

*D*—H⋯*A*	*D*—H	H⋯*A*	*D*⋯*A*	*D*—H⋯*A*
N2—H21⋯O2^i^	0.91 (2)	1.82 (2)	2.728 (2)	175 (2)
N2—H22⋯O1^ii^	0.91 (2)	1.78 (2)	2.691 (2)	172 (2)

Symmetry codes: (i) x, y+1, z; (ii) -x+1, -y, -z+1.

**Table 3:** Hydrogen-bond geometry (Å, °) for (III) *Cg2* is the centroid of the C6–C11 ring.

*D*—H⋯*A*	*D*—H	H⋯*A*	*D*⋯*A*	*D*—H⋯*A*
C3—H3*A*⋯O2^i^	0.97	2.56	3.272 (3)	130
C5—H5*A*⋯O1^ii^	0.97	2.56	3.469 (3)	156
N2—H21⋯O2^ii^	0.87 (2)	1.83 (2)	2.696 (3)	172 (3)
N2—H22⋯O1^iii^	0.88 (2)	1.83 (2)	2.700 (3)	172 (3)
C4—H4*B*⋯*Cg*2^iv^	0.97	2.59	3.473 (3)	152

Symmetry codes: (i) x, y+1, z; (ii) -x+2, -y+1, -z+1; (iii) -x+1, -y+1, -z+1; (iv) -x, -y, -z.

**Table 4:** Experimental details

	(I)	(II)	(III)
Crystal data			
Chemical formula	C_6_H_15_N_2_^+^·C_7_H_3_N_2_O_6_^−^	C_5_H_13_N_2_^+^·C_7_H_3_N_2_O_6_^−^	C_5_H_13_N_2_^+^·C_7_H_4_IO_2_^−^
*M*_r_	326.31	312.29	348.17
Crystal system, space group	Monoclinic, *C*2/*c*	Triclinic, *P*\overline{1}	Triclinic, *P*\overline{1}
Temperature (K)	293	293	293
*a*, *b*, *c* (Å)	19.362 (1), 8.6279 (7), 19.318 (1)	7.8023 (6), 10.3920 (8), 10.4770 (8)	6.2418 (4), 9.5465 (8), 12.5346 (9)
α, β, γ (°)	90, 97.261 (8), 90	73.578 (8), 74.289 (8), 71.828 (7)	110.708 (8), 90.235 (5), 101.559 (6)
*V* (Å^3^)	3201.3 (4)	758.49 (11)	682.19 (9)
*Z*	8	2	2
Radiation type	Mo *K*α	Mo *K*α	Mo *K*α
μ (mm^−1^)	0.11	0.11	2.34
Crystal size (mm)	0.46 × 0.28 × 0.24	0.48 × 0.48 × 0.44	0.48 × 0.24 × 0.2

Data collection			
Diffractometer	Oxford Diffraction Xcalibur	Oxford Diffraction Xcalibur	Oxford Diffraction Xcalibur
Absorption correction	Multi-scan (*CrysAlis RED*; Oxford Diffraction, 2009 ▸)	Multi-scan (*CrysAlis RED*; Oxford Diffraction, 2009 ▸)	Multi-scan (*CrysAlis RED*; Oxford Diffraction, 2009 ▸)
*T*_min_, *T*_max_	0.964, 0.974	0.948, 0.952	0.515, 0.626
No. of measured, independent and observed [*I* > 2σ(*I*)] reflections	6345, 2943, 1968	4819, 2774, 1935	4189, 2492, 2324
*R*_int_	0.016	0.010	0.011
(sin θ/λ)_max_ (Å^−1^)	0.602	0.602	0.602

Refinement			
*R*[*F* ^2^ > 2σ(*F* ^2^)], *wR*(*F* ^2^), *S*	0.048, 0.123, 1.04	0.044, 0.129, 1.03	0.020, 0.051, 1.11
No. of reflections	2943	2774	2492
No. of parameters	214	206	160
No. of restraints	2	2	2
H-atom treatment	H atoms treated by a mixture of independent and constrained refinement	H atoms treated by a mixture of independent and constrained refinement	H atoms treated by a mixture of independent and constrained refinement
Δρ_max_, Δρ_min_ (e Å^−3^)	0.20, −0.17	0.28, −0.15	0.37, −0.95

Computer programs: *CrysAlis CCD* (Oxford Diffraction, 2009 ▸), *CrysAlis RED* (Oxford Diffraction, 2009 ▸), *SHELXT* (Sheldrick, 2015*a* ▸), *Mercury* (Macrae *et al.*, 2020 ▸), *SHELXL2014* (Sheldrick, 2015*b* ▸), *PLATON* (Spek, 2020 ▸) and *publCIF* (Westrip, 2010 ▸).

## supplementary crystallographic information

### 4-Ethylpiperazin-1-ium 3,5-dinitrobenzoate (I) Crystal data

**Table:**

C_6_H_15_N_2_^+^·C_7_H_3_N_2_O_6_^−^	*F*(000) = 1376
*M**_r_* = 326.31	*D*_x_ = 1.354 Mg m^−^^3^
Monoclinic, *C*2/*c*	Mo *K*α radiation, λ = 0.71073 Å
Hall symbol: -C 2yc	Cell parameters from 6345 reflections
*a* = 19.362 (1) Å	θ = 2.6–25.4°
*b* = 8.6279 (7) Å	µ = 0.11 mm^−^^1^
*c* = 19.318 (1) Å	*T* = 293 K
β = 97.261 (8)°	Prism, orange
*V* = 3201.3 (4) Å^3^	0.46 × 0.28 × 0.24 mm
*Z* = 8	

### 4-Ethylpiperazin-1-ium 3,5-dinitrobenzoate (I) Data collection

**Table:**

Oxford Diffraction Xcalibur diffractometer	1968 reflections with *I* > 2σ(*I*)
Graphite monochromator	*R*_int_ = 0.016
ω scans	θ_max_ = 25.4°, θ_min_ = 2.6°
Absorption correction: multi-scan (CrysAlis RED; Oxford Diffraction, 2009)	*h* = −23→23
*T*_min_ = 0.964, *T*_max_ = 0.974	*k* = −10→10
6345 measured reflections	*l* = −9→23
2943 independent reflections	

### 4-Ethylpiperazin-1-ium 3,5-dinitrobenzoate (I) Refinement

**Table:**

Refinement on *F*^2^	Primary atom site location: structure-invariant direct methods
Least-squares matrix: full	Secondary atom site location: difference Fourier map
*R*[*F*^2^ > 2σ(*F*^2^)] = 0.048	Hydrogen site location: mixed
*wR*(*F*^2^) = 0.123	H atoms treated by a mixture of independent and constrained refinement
*S* = 1.04	*w* = 1/[σ^2^(*F*_o_^2^) + (0.0507*P*)^2^ + 1.8986*P*] where *P* = (*F*_o_^2^ + 2*F*_c_^2^)/3
2943 reflections	(Δ/σ)_max_ < 0.001
214 parameters	Δρ_max_ = 0.20 e Å^−^^3^
2 restraints	Δρ_min_ = −0.17 e Å^−^^3^
0 constraints	

### 4-Ethylpiperazin-1-ium 3,5-dinitrobenzoate (I) Special details

**Table:**

Geometry. All esds (except the esd in the dihedral angle between two l.s. planes) are estimated using the full covariance matrix. The cell esds are taken into account individually in the estimation of esds in distances, angles and torsion angles; correlations between esds in cell parameters are only used when they are defined by crystal symmetry. An approximate (isotropic) treatment of cell esds is used for estimating esds involving l.s. planes.

### 4-Ethylpiperazin-1-ium 3,5-dinitrobenzoate (I) Fractional atomic coordinates and isotropic or equivalent isotropic displacement parameters (Å^2^)

**Table:**

	*x*	*y*	*z*	*U*_iso_*/*U*_eq_	
C1	0.18587 (15)	0.3364 (3)	0.30396 (15)	0.0884 (9)	
H1A	0.232834	0.339426	0.326863	0.133*	
H1B	0.177587	0.424858	0.273903	0.133*	
H1C	0.154108	0.338102	0.338302	0.133*	
C2	0.17517 (12)	0.1925 (3)	0.26195 (13)	0.0655 (7)	
H2A	0.208261	0.190584	0.228215	0.079*	
H2B	0.184798	0.103891	0.292568	0.079*	
C3	0.08924 (11)	0.2969 (3)	0.17179 (12)	0.0533 (6)	
H3A	0.12338	0.291797	0.13919	0.064*	
H3B	0.092984	0.397926	0.194012	0.064*	
C4	0.01736 (11)	0.2779 (3)	0.13276 (13)	0.0589 (6)	
H4A	−0.017049	0.290226	0.164733	0.071*	
H4B	0.009231	0.357361	0.097152	0.071*	
C5	0.02593 (11)	0.0011 (3)	0.15297 (12)	0.0563 (6)	
H5A	0.023563	−0.099603	0.130507	0.068*	
H5B	−0.008248	0.003405	0.185592	0.068*	
C6	0.09733 (11)	0.0253 (3)	0.19161 (12)	0.0538 (6)	
H6A	0.106657	−0.054243	0.227061	0.065*	
H6B	0.131632	0.015195	0.159377	0.065*	
C7	0.22610 (9)	0.0114 (2)	0.00395 (10)	0.0389 (5)	
C8	0.25500 (10)	0.1070 (2)	0.05725 (10)	0.0431 (5)	
H8	0.226597	0.164062	0.08315	0.052*	
C9	0.32638 (10)	0.1168 (2)	0.07159 (10)	0.0449 (5)	
C10	0.37088 (10)	0.0395 (2)	0.03386 (11)	0.0468 (5)	
H10	0.418946	0.049122	0.043855	0.056*	
C11	0.34091 (9)	−0.0527 (2)	−0.01928 (11)	0.0442 (5)	
C12	0.26950 (9)	−0.0699 (2)	−0.03464 (10)	0.0428 (5)	
H12	0.250959	−0.135397	−0.070496	0.051*	
C13	0.14743 (10)	−0.0038 (3)	−0.01245 (10)	0.0440 (5)	
N1	0.10398 (8)	0.1771 (2)	0.22445 (8)	0.0478 (5)	
N2	0.00983 (8)	0.1234 (2)	0.09987 (9)	0.0502 (5)	
N3	0.35665 (12)	0.2143 (3)	0.13029 (11)	0.0657 (6)	
N4	0.38632 (9)	−0.1369 (2)	−0.06158 (12)	0.0612 (5)	
O1	0.11149 (7)	0.0962 (2)	0.01190 (8)	0.0621 (5)	
O2	0.12597 (7)	−0.1160 (2)	−0.05004 (9)	0.0653 (5)	
O3	0.31860 (12)	0.3056 (3)	0.15461 (11)	0.0980 (7)	
O4	0.41795 (10)	0.1972 (3)	0.15171 (10)	0.0987 (7)	
O5	0.44928 (7)	−0.1316 (2)	−0.04397 (10)	0.0829 (6)	
O6	0.35960 (9)	−0.2085 (3)	−0.11194 (11)	0.0962 (7)	
H21	0.0381 (14)	0.116 (4)	0.0660 (13)	0.115*	
H22	−0.0355 (10)	0.114 (4)	0.0790 (14)	0.115*	

### 4-Ethylpiperazin-1-ium 3,5-dinitrobenzoate (I) Atomic displacement parameters (Å^2^)

**Table:**

	*U*^11^	*U*^22^	*U*^33^	*U*^12^	*U*^13^	*U*^23^
C1	0.084 (2)	0.088 (2)	0.085 (2)	−0.0122 (16)	−0.0206 (15)	−0.0224 (17)
C2	0.0594 (14)	0.0728 (18)	0.0585 (14)	−0.0020 (12)	−0.0150 (11)	0.0000 (13)
C3	0.0511 (12)	0.0460 (13)	0.0597 (13)	−0.0019 (10)	−0.0053 (10)	−0.0028 (11)
C4	0.0477 (12)	0.0602 (15)	0.0652 (14)	0.0061 (11)	−0.0069 (11)	−0.0037 (13)
C5	0.0538 (13)	0.0543 (14)	0.0608 (14)	−0.0136 (11)	0.0072 (11)	−0.0063 (12)
C6	0.0546 (13)	0.0483 (14)	0.0563 (13)	−0.0017 (10)	−0.0015 (10)	0.0037 (12)
C7	0.0330 (9)	0.0390 (11)	0.0437 (11)	−0.0016 (8)	0.0007 (8)	0.0063 (10)
C8	0.0431 (11)	0.0413 (12)	0.0448 (11)	−0.0019 (9)	0.0053 (9)	0.0024 (10)
C9	0.0460 (11)	0.0423 (12)	0.0439 (11)	−0.0114 (10)	−0.0043 (9)	0.0027 (10)
C10	0.0331 (10)	0.0478 (12)	0.0565 (13)	−0.0072 (9)	−0.0057 (9)	0.0094 (11)
C11	0.0332 (10)	0.0446 (12)	0.0547 (12)	0.0004 (9)	0.0049 (9)	0.0032 (11)
C12	0.0370 (10)	0.0422 (12)	0.0479 (11)	−0.0039 (9)	−0.0002 (9)	0.0008 (10)
C13	0.0329 (10)	0.0542 (14)	0.0443 (11)	−0.0017 (10)	0.0026 (9)	0.0081 (11)
N1	0.0446 (9)	0.0524 (11)	0.0437 (10)	−0.0038 (8)	−0.0048 (8)	−0.0025 (9)
N2	0.0331 (9)	0.0683 (13)	0.0477 (10)	−0.0049 (9)	−0.0013 (7)	−0.0090 (10)
N3	0.0674 (14)	0.0690 (15)	0.0579 (12)	−0.0222 (12)	−0.0030 (11)	−0.0041 (12)
N4	0.0428 (11)	0.0591 (13)	0.0833 (14)	0.0009 (9)	0.0135 (10)	−0.0068 (12)
O1	0.0415 (8)	0.0734 (11)	0.0735 (11)	0.0069 (8)	0.0153 (7)	−0.0026 (9)
O2	0.0340 (8)	0.0748 (11)	0.0837 (11)	−0.0064 (8)	−0.0057 (7)	−0.0170 (10)
O3	0.1085 (17)	0.0929 (16)	0.0892 (15)	−0.0096 (13)	−0.0011 (12)	−0.0405 (13)
O4	0.0675 (12)	0.1305 (19)	0.0900 (14)	−0.0337 (12)	−0.0219 (10)	−0.0182 (13)
O5	0.0337 (9)	0.0929 (14)	0.1238 (16)	0.0008 (9)	0.0167 (9)	−0.0136 (12)
O6	0.0624 (11)	0.1236 (18)	0.1026 (14)	0.0096 (11)	0.0109 (10)	−0.0556 (14)

### 4-Ethylpiperazin-1-ium 3,5-dinitrobenzoate (I) Geometric parameters (Å, º)

**Table:**

C1—C2	1.484 (3)	C7—C8	1.381 (3)
C1—H1A	0.96	C7—C12	1.383 (3)
C1—H1B	0.96	C7—C13	1.522 (2)
C1—H1C	0.96	C8—C9	1.377 (3)
C2—N1	1.480 (3)	C8—H8	0.93
C2—H2A	0.97	C9—C10	1.370 (3)
C2—H2B	0.97	C9—N3	1.473 (3)
C3—N1	1.453 (3)	C10—C11	1.368 (3)
C3—C4	1.506 (3)	C10—H10	0.93
C3—H3A	0.97	C11—C12	1.385 (2)
C3—H3B	0.97	C11—N4	1.467 (3)
C4—N2	1.476 (3)	C12—H12	0.93
C4—H4A	0.97	C13—O1	1.238 (2)
C4—H4B	0.97	C13—O2	1.250 (2)
C5—N2	1.477 (3)	N2—H21	0.907 (17)
C5—C6	1.500 (3)	N2—H22	0.922 (17)
C5—H5A	0.97	N3—O3	1.213 (3)
C5—H5B	0.97	N3—O4	1.216 (2)
C6—N1	1.454 (3)	N4—O6	1.212 (2)
C6—H6A	0.97	N4—O5	1.224 (2)
C6—H6B	0.97		
			
C2—C1—H1A	109.5	C8—C7—C12	119.22 (17)
C2—C1—H1B	109.5	C8—C7—C13	120.47 (18)
H1A—C1—H1B	109.5	C12—C7—C13	120.31 (18)
C2—C1—H1C	109.5	C9—C8—C7	119.24 (19)
H1A—C1—H1C	109.5	C9—C8—H8	120.4
H1B—C1—H1C	109.5	C7—C8—H8	120.4
N1—C2—C1	113.6 (2)	C10—C9—C8	123.05 (19)
N1—C2—H2A	108.9	C10—C9—N3	118.16 (18)
C1—C2—H2A	108.9	C8—C9—N3	118.8 (2)
N1—C2—H2B	108.9	C11—C10—C9	116.52 (17)
C1—C2—H2B	108.9	C11—C10—H10	121.7
H2A—C2—H2B	107.7	C9—C10—H10	121.7
N1—C3—C4	111.11 (18)	C10—C11—C12	122.71 (19)
N1—C3—H3A	109.4	C10—C11—N4	118.60 (17)
C4—C3—H3A	109.4	C12—C11—N4	118.69 (18)
N1—C3—H3B	109.4	C7—C12—C11	119.23 (19)
C4—C3—H3B	109.4	C7—C12—H12	120.4
H3A—C3—H3B	108	C11—C12—H12	120.4
N2—C4—C3	110.43 (17)	O1—C13—O2	126.81 (18)
N2—C4—H4A	109.6	O1—C13—C7	117.20 (19)
C3—C4—H4A	109.6	O2—C13—C7	115.98 (18)
N2—C4—H4B	109.6	C3—N1—C6	109.69 (16)
C3—C4—H4B	109.6	C3—N1—C2	111.54 (17)
H4A—C4—H4B	108.1	C6—N1—C2	108.66 (17)
N2—C5—C6	110.30 (17)	C4—N2—C5	110.20 (17)
N2—C5—H5A	109.6	C4—N2—H21	110 (2)
C6—C5—H5A	109.6	C5—N2—H21	111 (2)
N2—C5—H5B	109.6	C4—N2—H22	108 (2)
C6—C5—H5B	109.6	C5—N2—H22	110.3 (19)
H5A—C5—H5B	108.1	H21—N2—H22	108 (3)
N1—C6—C5	111.54 (18)	O3—N3—O4	124.2 (2)
N1—C6—H6A	109.3	O3—N3—C9	117.8 (2)
C5—C6—H6A	109.3	O4—N3—C9	118.0 (2)
N1—C6—H6B	109.3	O6—N4—O5	123.5 (2)
C5—C6—H6B	109.3	O6—N4—C11	118.33 (18)
H6A—C6—H6B	108	O5—N4—C11	118.2 (2)
			
N1—C3—C4—N2	−57.7 (2)	C12—C7—C13—O2	15.6 (3)
N2—C5—C6—N1	57.5 (2)	C4—C3—N1—C6	58.2 (2)
C12—C7—C8—C9	−1.2 (3)	C4—C3—N1—C2	178.59 (18)
C13—C7—C8—C9	179.11 (18)	C5—C6—N1—C3	−58.3 (2)
C7—C8—C9—C10	2.1 (3)	C5—C6—N1—C2	179.54 (18)
C7—C8—C9—N3	−177.62 (17)	C1—C2—N1—C3	65.2 (3)
C8—C9—C10—C11	−1.2 (3)	C1—C2—N1—C6	−173.8 (2)
N3—C9—C10—C11	178.58 (18)	C3—C4—N2—C5	56.1 (2)
C9—C10—C11—C12	−0.6 (3)	C6—C5—N2—C4	−55.9 (2)
C9—C10—C11—N4	179.50 (18)	C10—C9—N3—O3	165.7 (2)
C8—C7—C12—C11	−0.5 (3)	C8—C9—N3—O3	−14.5 (3)
C13—C7—C12—C11	179.18 (18)	C10—C9—N3—O4	−15.0 (3)
C10—C11—C12—C7	1.5 (3)	C8—C9—N3—O4	164.7 (2)
N4—C11—C12—C7	−178.69 (18)	C10—C11—N4—O6	−174.2 (2)
C8—C7—C13—O1	16.0 (3)	C12—C11—N4—O6	5.9 (3)
C12—C7—C13—O1	−163.64 (18)	C10—C11—N4—O5	6.0 (3)
C8—C7—C13—O2	−164.73 (18)	C12—C11—N4—O5	−173.9 (2)

### 4-Ethylpiperazin-1-ium 3,5-dinitrobenzoate (I) Hydrogen-bond geometry (Å, º)

**Table:**

*D*—H···*A*	*D*—H	H···*A*	*D*···*A*	*D*—H···*A*
N2—H21···O1	0.91 (2)	1.88 (2)	2.768 (2)	168 (3)
N2—H22···O2^i^	0.92 (2)	1.77 (2)	2.684 (2)	171 (3)

Symmetry code: (i) −*x*, −*y*, −*z*.

### 4-Methylpiperazin-1-ium 3,5-dinitrobenzoate (II) Crystal data

**Table:**

C_5_H_13_N_2_^+^·C_7_H_3_N_2_O_6_^−^	*Z* = 2
*M**_r_* = 312.29	*F*(000) = 328
Triclinic, *P*1	*D*_x_ = 1.367 Mg m^−^^3^
Hall symbol: -P 1	Mo *K*α radiation, λ = 0.71073 Å
*a* = 7.8023 (6) Å	Cell parameters from 4819 reflections
*b* = 10.3920 (8) Å	θ = 2.6–25.4°
*c* = 10.4770 (8) Å	µ = 0.11 mm^−^^1^
α = 73.578 (8)°	*T* = 293 K
β = 74.289 (8)°	Prism, orange
γ = 71.828 (7)°	0.48 × 0.48 × 0.44 mm
*V* = 758.49 (11) Å^3^	

### 4-Methylpiperazin-1-ium 3,5-dinitrobenzoate (II) Data collection

**Table:**

Oxford Diffraction Xcalibur diffractometer	1935 reflections with *I* > 2σ(*I*)
Graphite monochromator	*R*_int_ = 0.010
ω scans	θ_max_ = 25.4°, θ_min_ = 2.6°
Absorption correction: multi-scan (CrysAlis RED; Oxford Diffraction, 2009)	*h* = −9→7
*T*_min_ = 0.948, *T*_max_ = 0.952	*k* = −12→8
4819 measured reflections	*l* = −12→12
2774 independent reflections	

### 4-Methylpiperazin-1-ium 3,5-dinitrobenzoate (II) Refinement

**Table:**

Refinement on *F*^2^	Primary atom site location: structure-invariant direct methods
Least-squares matrix: full	Secondary atom site location: difference Fourier map
*R*[*F*^2^ > 2σ(*F*^2^)] = 0.044	Hydrogen site location: mixed
*wR*(*F*^2^) = 0.129	H atoms treated by a mixture of independent and constrained refinement
*S* = 1.03	*w* = 1/[σ^2^(*F*_o_^2^) + (0.0593*P*)^2^ + 0.1428*P*] where *P* = (*F*_o_^2^ + 2*F*_c_^2^)/3
2774 reflections	(Δ/σ)_max_ < 0.001
206 parameters	Δρ_max_ = 0.28 e Å^−^^3^
2 restraints	Δρ_min_ = −0.15 e Å^−^^3^
0 constraints	

### 4-Methylpiperazin-1-ium 3,5-dinitrobenzoate (II) Special details

**Table:**

Geometry. All esds (except the esd in the dihedral angle between two l.s. planes) are estimated using the full covariance matrix. The cell esds are taken into account individually in the estimation of esds in distances, angles and torsion angles; correlations between esds in cell parameters are only used when they are defined by crystal symmetry. An approximate (isotropic) treatment of cell esds is used for estimating esds involving l.s. planes.

### 4-Methylpiperazin-1-ium 3,5-dinitrobenzoate (II) Fractional atomic coordinates and isotropic or equivalent isotropic displacement parameters (Å^2^)

**Table:**

	*x*	*y*	*z*	*U*_iso_*/*U*_eq_	
O1	0.4259 (3)	−0.25601 (14)	0.60539 (19)	0.1005 (6)	
O2	0.3187 (2)	−0.28191 (15)	0.44153 (17)	0.0950 (5)	
O3	0.2206 (3)	0.1945 (2)	0.7260 (2)	0.1219 (7)	
O4	0.1284 (3)	0.37901 (16)	0.5805 (2)	0.1139 (7)	
O5	0.0144 (3)	0.3389 (2)	0.16451 (19)	0.1237 (7)	
O6	0.0455 (3)	0.1363 (3)	0.1329 (2)	0.1293 (8)	
N3	0.1771 (3)	0.25321 (19)	0.6173 (2)	0.0806 (5)	
N4	0.0540 (3)	0.2122 (3)	0.1988 (2)	0.0877 (6)	
C6	0.2667 (2)	−0.05607 (17)	0.47269 (17)	0.0500 (4)	
C7	0.2608 (2)	0.02602 (17)	0.55809 (18)	0.0535 (4)	
H7	0.307549	−0.013627	0.637091	0.064*	
C8	0.1847 (2)	0.16762 (17)	0.52500 (19)	0.0569 (4)	
C9	0.1152 (2)	0.23142 (19)	0.4090 (2)	0.0625 (5)	
H9	0.064143	0.326656	0.38815	0.075*	
C10	0.1249 (2)	0.1477 (2)	0.32539 (19)	0.0606 (5)	
C11	0.1974 (2)	0.00621 (19)	0.35514 (18)	0.0568 (5)	
H11	0.199786	−0.047271	0.29663	0.068*	
C12	0.3453 (3)	−0.21207 (18)	0.5093 (2)	0.0606 (5)	
N1	0.4346 (2)	0.71225 (17)	0.02379 (16)	0.0702 (5)	
N2	0.4591 (3)	0.52702 (16)	0.28262 (17)	0.0687 (5)	
C1	0.3857 (4)	0.8479 (3)	−0.0671 (3)	0.1066 (9)	
H1A	0.493157	0.865009	−0.133097	0.16*	
H1B	0.338418	0.918742	−0.015173	0.16*	
H1C	0.293597	0.849066	−0.112598	0.16*	
C2	0.2725 (3)	0.6783 (2)	0.1169 (2)	0.0697 (5)	
H2A	0.184008	0.681477	0.06581	0.084*	
H2B	0.21612	0.74668	0.172715	0.084*	
C3	0.3197 (3)	0.5366 (2)	0.2067 (2)	0.0739 (6)	
H3A	0.209494	0.517861	0.270132	0.089*	
H3B	0.366896	0.467313	0.15165	0.089*	
C4	0.6227 (3)	0.5678 (2)	0.1885 (2)	0.0809 (6)	
H4A	0.684243	0.501297	0.131159	0.097*	
H4B	0.708662	0.568073	0.240126	0.097*	
C5	0.5641 (3)	0.7102 (2)	0.1016 (2)	0.0767 (6)	
H5A	0.50716	0.777105	0.15895	0.092*	
H5B	0.671413	0.736436	0.040146	0.092*	
H21	0.412 (3)	0.586 (2)	0.340 (2)	0.092*	
H22	0.494 (3)	0.4374 (18)	0.329 (2)	0.092*	

### 4-Methylpiperazin-1-ium 3,5-dinitrobenzoate (II) Atomic displacement parameters (Å^2^)

**Table:**

	*U*^11^	*U*^22^	*U*^33^	*U*^12^	*U*^13^	*U*^23^
O1	0.1400 (15)	0.0432 (8)	0.1110 (13)	0.0019 (8)	−0.0587 (12)	−0.0017 (8)
O2	0.1243 (14)	0.0579 (9)	0.0982 (12)	−0.0057 (8)	−0.0139 (10)	−0.0373 (9)
O3	0.188 (2)	0.0895 (13)	0.1115 (15)	−0.0329 (13)	−0.0574 (15)	−0.0344 (12)
O4	0.1277 (14)	0.0523 (10)	0.1664 (18)	−0.0016 (9)	−0.0407 (13)	−0.0441 (11)
O5	0.1127 (14)	0.1017 (15)	0.1015 (13)	0.0206 (11)	−0.0330 (11)	0.0208 (11)
O6	0.1450 (19)	0.149 (2)	0.0972 (14)	−0.0207 (14)	−0.0625 (13)	−0.0148 (14)
N3	0.0813 (12)	0.0582 (11)	0.1069 (16)	−0.0145 (9)	−0.0177 (11)	−0.0297 (11)
N4	0.0675 (12)	0.0988 (16)	0.0703 (13)	−0.0021 (10)	−0.0176 (9)	0.0047 (12)
C6	0.0438 (9)	0.0429 (9)	0.0544 (10)	−0.0084 (7)	−0.0007 (7)	−0.0086 (8)
C7	0.0489 (9)	0.0469 (10)	0.0578 (10)	−0.0098 (7)	−0.0088 (8)	−0.0053 (8)
C8	0.0514 (10)	0.0439 (10)	0.0721 (12)	−0.0111 (7)	−0.0067 (9)	−0.0140 (9)
C9	0.0484 (10)	0.0435 (10)	0.0788 (13)	−0.0071 (7)	−0.0067 (9)	0.0018 (9)
C10	0.0448 (10)	0.0617 (12)	0.0590 (11)	−0.0073 (8)	−0.0074 (8)	0.0023 (9)
C11	0.0487 (10)	0.0597 (11)	0.0562 (10)	−0.0122 (8)	−0.0013 (8)	−0.0142 (9)
C12	0.0630 (11)	0.0420 (10)	0.0627 (12)	−0.0068 (8)	0.0020 (9)	−0.0110 (9)
N1	0.0768 (11)	0.0689 (11)	0.0543 (9)	−0.0116 (8)	−0.0144 (8)	−0.0036 (8)
N2	0.0975 (13)	0.0401 (8)	0.0587 (10)	0.0023 (8)	−0.0235 (9)	−0.0102 (7)
C1	0.123 (2)	0.0942 (19)	0.0786 (16)	−0.0234 (15)	−0.0293 (15)	0.0223 (14)
C2	0.0695 (13)	0.0647 (12)	0.0737 (13)	−0.0130 (10)	−0.0253 (10)	−0.0075 (10)
C3	0.0870 (15)	0.0604 (12)	0.0751 (13)	−0.0214 (10)	−0.0180 (11)	−0.0115 (10)
C4	0.0717 (14)	0.0743 (14)	0.0922 (16)	0.0063 (11)	−0.0325 (12)	−0.0237 (12)
C5	0.0638 (12)	0.0790 (14)	0.0780 (14)	−0.0189 (10)	−0.0099 (11)	−0.0061 (11)

### 4-Methylpiperazin-1-ium 3,5-dinitrobenzoate (II) Geometric parameters (Å, º)

**Table:**

O1—C12	1.233 (2)	N1—C5	1.452 (3)
O2—C12	1.241 (2)	N1—C1	1.464 (3)
O3—N3	1.213 (3)	N2—C3	1.477 (3)
O4—N3	1.219 (2)	N2—C4	1.483 (3)
O5—N4	1.224 (3)	N2—H21	0.906 (16)
O6—N4	1.212 (3)	N2—H22	0.913 (16)
N3—C8	1.467 (3)	C1—H1A	0.96
N4—C10	1.475 (3)	C1—H1B	0.96
C6—C7	1.384 (2)	C1—H1C	0.96
C6—C11	1.386 (2)	C2—C3	1.502 (3)
C6—C12	1.519 (2)	C2—H2A	0.97
C7—C8	1.384 (2)	C2—H2B	0.97
C7—H7	0.93	C3—H3A	0.97
C8—C9	1.374 (3)	C3—H3B	0.97
C9—C10	1.373 (3)	C4—C5	1.506 (3)
C9—H9	0.93	C4—H4A	0.97
C10—C11	1.377 (2)	C4—H4B	0.97
C11—H11	0.93	C5—H5A	0.97
N1—C2	1.446 (2)	C5—H5B	0.97
			
O3—N3—O4	123.6 (2)	C3—N2—H22	108.4 (15)
O3—N3—C8	117.95 (18)	C4—N2—H22	109.2 (14)
O4—N3—C8	118.4 (2)	H21—N2—H22	111 (2)
O6—N4—O5	124.2 (2)	N1—C1—H1A	109.5
O6—N4—C10	117.9 (2)	N1—C1—H1B	109.5
O5—N4—C10	117.8 (2)	H1A—C1—H1B	109.5
C7—C6—C11	118.91 (16)	N1—C1—H1C	109.5
C7—C6—C12	120.13 (16)	H1A—C1—H1C	109.5
C11—C6—C12	120.95 (17)	H1B—C1—H1C	109.5
C6—C7—C8	119.47 (17)	N1—C2—C3	111.25 (17)
C6—C7—H7	120.3	N1—C2—H2A	109.4
C8—C7—H7	120.3	C3—C2—H2A	109.4
C9—C8—C7	122.55 (18)	N1—C2—H2B	109.4
C9—C8—N3	118.53 (17)	C3—C2—H2B	109.4
C7—C8—N3	118.92 (18)	H2A—C2—H2B	108
C10—C9—C8	116.73 (16)	N2—C3—C2	110.67 (16)
C10—C9—H9	121.6	N2—C3—H3A	109.5
C8—C9—H9	121.6	C2—C3—H3A	109.5
C9—C10—C11	122.66 (18)	N2—C3—H3B	109.5
C9—C10—N4	118.52 (19)	C2—C3—H3B	109.5
C11—C10—N4	118.8 (2)	H3A—C3—H3B	108.1
C10—C11—C6	119.67 (18)	N2—C4—C5	109.78 (16)
C10—C11—H11	120.2	N2—C4—H4A	109.7
C6—C11—H11	120.2	C5—C4—H4A	109.7
O1—C12—O2	126.92 (18)	N2—C4—H4B	109.7
O1—C12—C6	116.25 (18)	C5—C4—H4B	109.7
O2—C12—C6	116.81 (19)	H4A—C4—H4B	108.2
C2—N1—C5	108.81 (15)	N1—C5—C4	110.61 (18)
C2—N1—C1	110.74 (18)	N1—C5—H5A	109.5
C5—N1—C1	110.91 (18)	C4—C5—H5A	109.5
C3—N2—C4	110.66 (16)	N1—C5—H5B	109.5
C3—N2—H21	110.0 (15)	C4—C5—H5B	109.5
C4—N2—H21	107.1 (14)	H5A—C5—H5B	108.1
			
C11—C6—C7—C8	0.5 (2)	C9—C10—C11—C6	−1.0 (3)
C12—C6—C7—C8	−178.42 (15)	N4—C10—C11—C6	179.18 (15)
C6—C7—C8—C9	−0.6 (3)	C7—C6—C11—C10	0.2 (2)
C6—C7—C8—N3	179.23 (15)	C12—C6—C11—C10	179.17 (15)
O3—N3—C8—C9	172.3 (2)	C7—C6—C12—O1	−10.5 (2)
O4—N3—C8—C9	−8.0 (3)	C11—C6—C12—O1	170.56 (17)
O3—N3—C8—C7	−7.5 (3)	C7—C6—C12—O2	168.17 (16)
O4—N3—C8—C7	172.18 (18)	C11—C6—C12—O2	−10.8 (2)
C7—C8—C9—C10	−0.2 (3)	C5—N1—C2—C3	−60.3 (2)
N3—C8—C9—C10	−179.99 (15)	C1—N1—C2—C3	177.52 (19)
C8—C9—C10—C11	1.0 (3)	C4—N2—C3—C2	−54.0 (2)
C8—C9—C10—N4	−179.21 (15)	N1—C2—C3—N2	57.2 (2)
O6—N4—C10—C9	−172.5 (2)	C3—N2—C4—C5	54.9 (2)
O5—N4—C10—C9	10.3 (3)	C2—N1—C5—C4	61.4 (2)
O6—N4—C10—C11	7.3 (3)	C1—N1—C5—C4	−176.53 (19)
O5—N4—C10—C11	−169.91 (18)	N2—C4—C5—N1	−59.1 (2)

### 4-Methylpiperazin-1-ium 3,5-dinitrobenzoate (II) Hydrogen-bond geometry (Å, º)

**Table:**

*D*—H···*A*	*D*—H	H···*A*	*D*···*A*	*D*—H···*A*
N2—H21···O2^i^	0.91 (2)	1.82 (2)	2.728 (2)	175 (2)
N2—H22···O1^ii^	0.91 (2)	1.78 (2)	2.691 (2)	172 (2)

Symmetry codes: (i) *x*, *y*+1, *z*; (ii) −*x*+1, −*y*, −*z*+1.

### 4-Methylpiperazin-1-ium 4-iodobenzoate (III) Crystal data

**Table:**

C_5_H_13_N_2_^+^·C_7_H_4_IO_2_^−^	*Z* = 2
*M**_r_* = 348.17	*F*(000) = 344
Triclinic, *P*1	*D*_x_ = 1.695 Mg m^−^^3^
Hall symbol: -P 1	Mo *K*α radiation, λ = 0.71073 Å
*a* = 6.2418 (4) Å	Cell parameters from 4189 reflections
*b* = 9.5465 (8) Å	θ = 3.3–25.4°
*c* = 12.5346 (9) Å	µ = 2.34 mm^−^^1^
α = 110.708 (8)°	*T* = 293 K
β = 90.235 (5)°	Rods, colourless
γ = 101.559 (6)°	0.48 × 0.24 × 0.2 mm
*V* = 682.19 (9) Å^3^	

### 4-Methylpiperazin-1-ium 4-iodobenzoate (III) Data collection

**Table:**

Oxford Diffraction Xcalibur diffractometer	2324 reflections with *I* > 2σ(*I*)
Graphite monochromator	*R*_int_ = 0.011
ω scans	θ_max_ = 25.4°, θ_min_ = 3.3°
Absorption correction: multi-scan (CrysAlis RED; Oxford Diffraction, 2009)	*h* = −7→7
*T*_min_ = 0.515, *T*_max_ = 0.626	*k* = −11→11
4189 measured reflections	*l* = −15→14
2492 independent reflections	

### 4-Methylpiperazin-1-ium 4-iodobenzoate (III) Refinement

**Table:**

Refinement on *F*^2^	Primary atom site location: structure-invariant direct methods
Least-squares matrix: full	Secondary atom site location: difference Fourier map
*R*[*F*^2^ > 2σ(*F*^2^)] = 0.020	Hydrogen site location: mixed
*wR*(*F*^2^) = 0.051	H atoms treated by a mixture of independent and constrained refinement
*S* = 1.11	*w* = 1/[σ^2^(*F*_o_^2^) + (0.0289*P*)^2^ + 0.2845*P*] where *P* = (*F*_o_^2^ + 2*F*_c_^2^)/3
2492 reflections	(Δ/σ)_max_ < 0.001
160 parameters	Δρ_max_ = 0.37 e Å^−^^3^
2 restraints	Δρ_min_ = −0.95 e Å^−^^3^
0 constraints	

### 4-Methylpiperazin-1-ium 4-iodobenzoate (III) Special details

**Table:**

Geometry. All esds (except the esd in the dihedral angle between two l.s. planes) are estimated using the full covariance matrix. The cell esds are taken into account individually in the estimation of esds in distances, angles and torsion angles; correlations between esds in cell parameters are only used when they are defined by crystal symmetry. An approximate (isotropic) treatment of cell esds is used for estimating esds involving l.s. planes.

### 4-Methylpiperazin-1-ium 4-iodobenzoate (III) Fractional atomic coordinates and isotropic or equivalent isotropic displacement parameters (Å^2^)

**Table:**

	*x*	*y*	*z*	*U*_iso_*/*U*_eq_	
C1	1.2514 (5)	0.7762 (5)	0.1396 (3)	0.0712 (11)	
H1A	1.242455	0.690853	0.068934	0.107*	
H1B	1.283901	0.870031	0.124947	0.107*	
H1C	1.365551	0.776131	0.19115	0.107*	
C2	1.0513 (4)	0.8911 (3)	0.2981 (2)	0.0429 (6)	
H2A	1.085517	0.986567	0.284191	0.051*	
H2B	1.167221	0.892502	0.350502	0.051*	
C3	0.8371 (4)	0.8786 (3)	0.3512 (2)	0.0428 (6)	
H3A	0.847548	0.963711	0.423379	0.051*	
H3B	0.722435	0.883465	0.301016	0.051*	
C4	0.7745 (4)	0.6017 (3)	0.2629 (2)	0.0432 (6)	
H4A	0.656473	0.596833	0.210215	0.052*	
H4B	0.745692	0.506984	0.277895	0.052*	
C5	0.9887 (4)	0.6188 (3)	0.2099 (2)	0.0424 (6)	
H5A	1.10447	0.614721	0.259906	0.051*	
H5B	0.980005	0.534325	0.137531	0.051*	
C6	0.6076 (4)	0.2567 (3)	0.3636 (2)	0.0296 (5)	
C7	0.6850 (4)	0.1909 (3)	0.2577 (2)	0.0339 (5)	
H7	0.812059	0.153084	0.253927	0.041*	
C8	0.5770 (4)	0.1804 (3)	0.1574 (2)	0.0355 (5)	
H8	0.629598	0.134593	0.086951	0.043*	
C9	0.3881 (4)	0.2396 (3)	0.1635 (2)	0.0312 (5)	
C10	0.3100 (4)	0.3057 (3)	0.2684 (2)	0.0332 (5)	
H10	0.184232	0.345025	0.272511	0.04*	
C11	0.4189 (4)	0.3135 (3)	0.3679 (2)	0.0336 (5)	
H11	0.364548	0.357276	0.438151	0.04*	
C12	0.7285 (4)	0.2674 (3)	0.4722 (2)	0.0345 (5)	
I1	0.22668 (3)	0.23210 (2)	0.01333 (2)	0.03947 (7)	
N1	1.0417 (3)	0.7631 (3)	0.19094 (18)	0.0384 (5)	
N2	0.7815 (3)	0.7327 (3)	0.37083 (19)	0.0393 (5)	
O1	0.6295 (3)	0.2895 (3)	0.56080 (16)	0.0567 (6)	
O2	0.9237 (3)	0.2528 (3)	0.46569 (16)	0.0467 (5)	
H21	0.876 (4)	0.728 (4)	0.420 (2)	0.056*	
H22	0.653 (4)	0.728 (4)	0.400 (3)	0.056*	

### 4-Methylpiperazin-1-ium 4-iodobenzoate (III) Atomic displacement parameters (Å^2^)

**Table:**

	*U*^11^	*U*^22^	*U*^33^	*U*^12^	*U*^13^	*U*^23^
C1	0.0331 (15)	0.135 (3)	0.056 (2)	0.0112 (18)	0.0100 (14)	0.051 (2)
C2	0.0390 (14)	0.0381 (14)	0.0506 (16)	−0.0024 (11)	−0.0094 (12)	0.0208 (12)
C3	0.0459 (14)	0.0405 (14)	0.0395 (14)	0.0171 (12)	−0.0055 (11)	0.0075 (12)
C4	0.0434 (14)	0.0365 (14)	0.0484 (16)	−0.0033 (11)	−0.0035 (12)	0.0204 (12)
C5	0.0455 (15)	0.0428 (14)	0.0373 (14)	0.0159 (12)	0.0009 (11)	0.0092 (12)
C6	0.0241 (10)	0.0347 (12)	0.0346 (12)	0.0059 (9)	0.0019 (9)	0.0183 (10)
C7	0.0272 (11)	0.0423 (13)	0.0378 (13)	0.0142 (10)	0.0040 (9)	0.0174 (11)
C8	0.0362 (13)	0.0404 (13)	0.0311 (12)	0.0142 (10)	0.0056 (10)	0.0110 (11)
C9	0.0291 (11)	0.0343 (12)	0.0326 (12)	0.0050 (9)	−0.0031 (9)	0.0162 (10)
C10	0.0239 (11)	0.0430 (13)	0.0380 (13)	0.0108 (10)	0.0029 (9)	0.0193 (11)
C11	0.0299 (11)	0.0446 (14)	0.0321 (12)	0.0117 (10)	0.0066 (9)	0.0186 (11)
C12	0.0258 (11)	0.0471 (14)	0.0354 (13)	0.0087 (10)	0.0017 (9)	0.0203 (11)
I1	0.03737 (10)	0.05212 (12)	0.03188 (10)	0.01315 (7)	−0.00148 (7)	0.01691 (8)
N1	0.0269 (10)	0.0595 (14)	0.0346 (11)	0.0059 (9)	0.0023 (8)	0.0260 (10)
N2	0.0279 (10)	0.0639 (14)	0.0327 (11)	0.0124 (10)	0.0036 (8)	0.0241 (11)
O1	0.0371 (10)	0.1100 (18)	0.0370 (11)	0.0261 (11)	0.0097 (8)	0.0379 (12)
O2	0.0319 (9)	0.0780 (14)	0.0400 (10)	0.0216 (9)	0.0035 (8)	0.0278 (10)

### 4-Methylpiperazin-1-ium 4-iodobenzoate (III) Geometric parameters (Å, º)

**Table:**

C1—N1	1.464 (3)	C5—H5B	0.97
C1—H1A	0.96	C6—C11	1.386 (3)
C1—H1B	0.96	C6—C7	1.388 (3)
C1—H1C	0.96	C6—C12	1.514 (3)
C2—N1	1.453 (4)	C7—C8	1.386 (4)
C2—C3	1.498 (4)	C7—H7	0.93
C2—H2A	0.97	C8—C9	1.398 (3)
C2—H2B	0.97	C8—H8	0.93
C3—N2	1.474 (4)	C9—C10	1.381 (3)
C3—H3A	0.97	C9—I1	2.103 (2)
C3—H3B	0.97	C10—C11	1.389 (3)
C4—N2	1.475 (4)	C10—H10	0.93
C4—C5	1.501 (4)	C11—H11	0.93
C4—H4A	0.97	C12—O1	1.246 (3)
C4—H4B	0.97	C12—O2	1.254 (3)
C5—N1	1.454 (4)	N2—H21	0.874 (18)
C5—H5A	0.97	N2—H22	0.881 (18)
			
N1—C1—H1A	109.5	C11—C6—C7	118.6 (2)
N1—C1—H1B	109.5	C11—C6—C12	120.8 (2)
H1A—C1—H1B	109.5	C7—C6—C12	120.6 (2)
N1—C1—H1C	109.5	C8—C7—C6	121.5 (2)
H1A—C1—H1C	109.5	C8—C7—H7	119.3
H1B—C1—H1C	109.5	C6—C7—H7	119.3
N1—C2—C3	110.8 (2)	C7—C8—C9	119.1 (2)
N1—C2—H2A	109.5	C7—C8—H8	120.4
C3—C2—H2A	109.5	C9—C8—H8	120.4
N1—C2—H2B	109.5	C10—C9—C8	119.9 (2)
C3—C2—H2B	109.5	C10—C9—I1	120.07 (17)
H2A—C2—H2B	108.1	C8—C9—I1	120.00 (18)
N2—C3—C2	110.0 (2)	C9—C10—C11	120.1 (2)
N2—C3—H3A	109.7	C9—C10—H10	119.9
C2—C3—H3A	109.7	C11—C10—H10	119.9
N2—C3—H3B	109.7	C6—C11—C10	120.8 (2)
C2—C3—H3B	109.7	C6—C11—H11	119.6
H3A—C3—H3B	108.2	C10—C11—H11	119.6
N2—C4—C5	110.2 (2)	O1—C12—O2	124.5 (2)
N2—C4—H4A	109.6	O1—C12—C6	118.7 (2)
C5—C4—H4A	109.6	O2—C12—C6	116.7 (2)
N2—C4—H4B	109.6	C2—N1—C5	110.3 (2)
C5—C4—H4B	109.6	C2—N1—C1	110.5 (2)
H4A—C4—H4B	108.1	C5—N1—C1	109.6 (2)
N1—C5—C4	111.1 (2)	C3—N2—C4	110.6 (2)
N1—C5—H5A	109.4	C3—N2—H21	112 (2)
C4—C5—H5A	109.4	C4—N2—H21	108 (2)
N1—C5—H5B	109.4	C3—N2—H22	108 (2)
C4—C5—H5B	109.4	C4—N2—H22	111 (2)
H5A—C5—H5B	108	H21—N2—H22	108 (3)
			
N1—C2—C3—N2	58.1 (3)	C9—C10—C11—C6	0.6 (4)
N2—C4—C5—N1	−56.7 (3)	C11—C6—C12—O1	18.7 (4)
C11—C6—C7—C8	−0.4 (4)	C7—C6—C12—O1	−161.8 (2)
C12—C6—C7—C8	−179.8 (2)	C11—C6—C12—O2	−161.4 (2)
C6—C7—C8—C9	1.0 (4)	C7—C6—C12—O2	18.0 (4)
C7—C8—C9—C10	−0.8 (4)	C3—C2—N1—C5	−58.7 (3)
C7—C8—C9—I1	177.94 (18)	C3—C2—N1—C1	180.0 (2)
C8—C9—C10—C11	0.0 (4)	C4—C5—N1—C2	58.0 (3)
I1—C9—C10—C11	−178.72 (18)	C4—C5—N1—C1	179.9 (2)
C7—C6—C11—C10	−0.4 (4)	C2—C3—N2—C4	−56.8 (3)
C12—C6—C11—C10	179.0 (2)	C5—C4—N2—C3	56.1 (3)

### 4-Methylpiperazin-1-ium 4-iodobenzoate (III) Hydrogen-bond geometry (Å, º)

*Cg2* is the centroid of the C6–C11 ring.

**Table:**

*D*—H···*A*	*D*—H	H···*A*	*D*···*A*	*D*—H···*A*
C3—H3*A*···O2^i^	0.97	2.56	3.272 (3)	130
C5—H5*A*···O1^ii^	0.97	2.56	3.469 (3)	156
N2—H21···O2^ii^	0.87 (2)	1.83 (2)	2.696 (3)	172 (3)
N2—H22···O1^iii^	0.88 (2)	1.83 (2)	2.700 (3)	172 (3)
C4—H4*B*···*Cg*2^iv^	0.97	2.59	3.473 (3)	152

Symmetry codes: (i) *x*, *y*+1, *z*; (ii) −*x*+2, −*y*+1, −*z*+1; (iii) −*x*+1, −*y*+1, −*z*+1; (iv) −*x*, −*y*, −*z*.
